# Exploring the relative importance of the factors associated with menopausal symptoms using a random forest model: a cross-sectional study

**DOI:** 10.4069/whn.2025.08.12

**Published:** 2025-09-30

**Authors:** Meejung Chin, Sowon Hahn, Yeon Soo Kim, Young Hye Kwon, Yeon-Hwan Park, Younghwan Choi, Gahye Kim, Youjin Kim, Inju Lee, Hyun Jeong Yoon, Hanjin Bae

**Affiliations:** 1 Department of Child Development and Family Studies, Seoul National University, Seoul, Korea; 2 Integrated Major in Regional Studies and Spatial Analytics, Seoul National University, Seoul, Korea; 3 Department of Psychology, Seoul National University, Seoul, Korea; 4 Department of Physical Education, College of Education, Seoul National University, Seoul, Korea; 5 Institute of Sport Science, Seoul National University, Seoul, Korea; 6 Department of Food and Nutrition, Seoul National University, Seoul, Korea; 7 College of Nursing, The Research Institute of Nursing Science, Seoul National University, Seoul, Korea; 8 College of Nursing, Eulji University, Uijeongbu, Korea

**Keywords:** Digital health, Menopause, Middle aged, Random forest, Women

## Abstract

**Purpose:**

This study aimed to identify key factors associated with menopausal symptoms among middle-aged women by examining a comprehensive set of physical, psychological, and lifestyle variables.

**Methods:**

A cross-sectional study was conducted with 94 women aged 45 to 55 years in Seoul, South Korea. Data were collected through physical assessments, self-reported questionnaires, and monitoring using a wearable device (Fitbit Charge 5, Fitbit Inc.). The Menopause Rating Scale was used to assess symptom severity, with scores dichotomized into no/mild symptoms (0–8) and moderate/severe symptoms (9–44). Random forest analysis was applied to evaluate the relative importance of various factors in relation to menopausal symptoms.

**Results:**

Fourteen significant predictors were identified from an initial set of 57 variables using recursive feature elimination with cross-validation. The final random forest model achieved balanced predictive performance, with an accuracy of 74.1%, an area under the curve of 75.7%, sensitivity of 78.9%, and specificity of 62.5%. Age emerged as the most influential predictor, followed by psychological well-being and loneliness as the second and third most important factors. Among physical characteristics, relative grip strength (fourth) and body fat percentage (fifth) were significant predictors. Lifestyle factors, including moderate physical activity (sixth) and health-conscious dietary behaviors (seventh), showed moderate importance, while socioeconomic factors demonstrated lower importance.

**Conclusion:**

The findings highlight the multifaceted nature of menopausal symptoms and suggest that effective management strategies should incorporate physical, psychological, and lifestyle interventions. These results provide evidence for developing comprehensive digital healthcare applications that incorporate monitoring and intervention features across multiple domains for effective menopausal symptom management.

## Introduction

Menopause is accompanied by a wide range of symptoms that significantly affect the physical and psychological health of middle-aged women [1]. Women in the menopausal transition often experience complex symptoms such as hot flashes, sleep disturbances, anxiety, and mood swings [2,3]. Research has demonstrated that these symptoms reduce quality of life and interfere with daily functioning [4]. The physical and psychological changes associated with menopause can also hinder health behaviors in middle-aged women. Reduced muscle strength and flexibility limit regular exercise [5], while depression and anxiety during this period may lead to unhealthy behaviors such as binge eating or reduced physical activity [6]. These challenges highlight the importance of supporting middle-aged women in adapting to menopausal changes and managing symptoms effectively.

Research across multiple disciplines has investigated the factors contributing to variability in symptoms, which can be grouped into physical, psychological, and lifestyle domains. Body composition, muscle strength, and physical fitness significantly affect symptom severity and management [7-9]. A higher body fat percentage has been strongly linked to more severe menopausal symptoms, with evidence showing that body fat can account for nearly half of the variance in total symptoms among women in the menopausal transition [10]. Conversely, studies suggest that increasing muscle mass and improving relative grip strength may help reduce symptom severity [7,11]. Age, reproductive health factors, and chronic conditions also play critical roles in shaping the onset and intensity of symptoms [12]. For instance, older age and reproductive factors such as later age at menarche and multiple pregnancies have been associated with more severe menopausal symptoms [13,14].

Furthermore, psychological well-being and social relationships play crucial roles in how women experience and cope with symptoms. For example, research has reported that menopausal symptoms can worsen psychological well-being [15]. However, psychological well-being, which includes autonomy, personal growth, and positive relationships, can affect psychological symptoms like anxiety and depression during menopause [16,17]. Similarly, loneliness has been shown to worsen menopausal symptoms, with women reporting more severe physical and emotional symptoms when feeling socially isolated [18]. Furthermore, social support and family relationships play a critical role in mitigating these challenges, with stronger family ties and social networks associated with fewer and less severe menopausal symptoms [19].

Physical activity levels, dietary patterns, and health-related behaviors are additional factors that influence the severity of menopausal symptoms. Regular physical activity has consistently been shown to reduce symptom severity, underscoring the importance of maintaining physical health during menopause [7]. Dietary habits characterized by high consumption of saturated fats, processed foods, and desserts have been linked to psychological, vasomotor, and somatic symptoms in postmenopausal women [20]. Unhealthy behaviors such as smoking and excessive alcohol intake are also associated with more severe symptoms [21]. Socioeconomic status further affects symptom severity, with women of lower educational attainment and lower household income reporting higher rates of sleep disturbances and depressed mood [22,23]. These findings underscore the multifactorial nature of menopausal symptoms and highlight the need for comprehensive research that integrates multiple domains to guide more effective management strategies.

Digital healthcare solutions have recently emerged as a promising strategy for addressing these diverse challenges. One study found that 8 weeks of using digital healthcare applications that provided personalized information, exercise coaching, and management of appointments and medications significantly reduced menopausal symptoms by improving physical quality of life and alleviating psychological distress through increased accessibility to treatment [24]. Similarly, a study of 1,900 women using the “Health & Her” app demonstrated that consistent symptom tracking and sustained engagement over 2 months resulted in significant reductions in menopausal symptoms [25]. Maximizing the impact of digital healthcare applications requires the careful selection of intervention strategies and monitoring variables. However, a review of 35 menopause-focused digital healthcare applications revealed that most lacked a scientific evidence base [26]. Given the multifaceted nature of menopausal symptoms, identifying the relative explanatory power of associated factors is essential for designing efficient and effective evidence-based health interventions.

In response to these challenges, our study adopted a multifaceted data collection approach. Participants underwent comprehensive physical assessments during in-person visits, including measurements of body mass index (BMI), muscle strength, and cardiorespiratory endurance. Detailed information on sociodemographic characteristics, social relationships, psychological factors, and dietary habits was collected through self-reported questionnaires. Additionally, physical activity patterns were monitored using wearable fitness trackers over an eight-day period. To analyze these data, we employed random forest analysis, an advanced machine learning technique that enables the simultaneous examination of variables across multiple domains and determines their relative importance [27]. Although recent studies have applied machine learning methods to analyze menopausal symptoms, they have generally focused on limited sets of variables rather than comprehensively addressing physical, psychological, and lifestyle factors together [28,29].

This study aimed to identify the key predictors of menopausal symptoms among middle-aged women by comprehensively analyzing physical, psychological, and lifestyle factors using random forest analysis. By leveraging this machine learning approach, we sought to determine the relative importance of multiple factors and their relationships with symptom severity, thereby providing evidence-based guidance for the development of digital healthcare applications. The findings of this study have significant implications for targeted interventions and digital health programs, supporting a more holistic and personalized approach to menopausal symptom management.

## Methods

**Ethics statement:** This study was approved by the Institutional Review Board of Seoul National University (No. 2302/002-008). Informed consent was obtained from the participants.

### Study design

This study employed a cross-sectional quantitative design with a correlational analysis approach to examine relationships between multiple predictor variables and menopausal symptoms. Data were collected as part of the project “Development of multidisciplinary well-aging indicators and digital application for menopause symptom monitoring of middle-aged women,” which was supported by Seoul National University from September 1, 2022, to April 30, 2024. Reporting of the study followed the STROBE (Strengthening the Reporting of Observational Studies in Epidemiology) guidelines (https://www.strobe-statement.org/).

### Participants

The study targeted women aged 45 to 55 years, corresponding to the average age range of menopausal transition in Korean women, in order to examine the relationship between menopausal symptoms and well-aging. Exclusion criteria were: (1) current use of sleep medications (as sleep quality was a key study variable), (2) inability to attend in-person physical assessments at the laboratory, (3) use of a non-Android smartphone (incompatible with the wearable device), and (4) insufficient Korean proficiency to complete self-administered questionnaires. Participants were recruited from February 14 to March 22, 2023, through recruitment announcements at public institutions (local government offices, health centers, and other community institutions) in Seoul, South Korea, and through advertisements on online community platforms.

The required sample size was estimated using G*Power 3.1.9.7 (Heinrich Heine University Düsseldorf, Düsseldorf, Germany), with an effect size of 0.3, significance level (α) of .05, and statistical power (1−β) of .80. This calculation indicated that a minimum of 82 participants was needed. In accordance with common practice in nursing research, where power is typically set between 0.80 and 0.95, we selected a power level of 0.80 [30]. The effect size was set at 0.3 (small-to-medium), based on prior studies examining the relationship between menopausal symptom status and physical capacity [31]. To account for possible dropouts and data quality issues during the 8-day wearable device monitoring period (e.g., non-adherence to wearing the device, device malfunction [32]), we aimed to recruit 30% more participants than the required minimum, targeting 106 participants. A total of 100 women were recruited. Of these, three were excluded for using sleep medications, two did not meet the age criterion, and one had incomplete Menopause Rating Scale (MRS) data, leaving a final sample of 94 participants. Although the original study design planned to compare women with and without menopausal symptoms, we ultimately used random forest analysis because it allows for effective handling of multiple predictors in relatively small samples and is well-suited for identifying complex patterns in the data.

### Measurements

Data were collected through self-reported questionnaires and objective physical assessments. Self-reported measures included menopausal symptoms, psychological factors, lifestyle behaviors, and general characteristics, while objective assessments consisted of body measurements and physical fitness testing.

#### Menopausal symptoms

Menopausal symptoms were measured using the Korean version [33] of the MRS [34]. This scale consists of 11 items covering physical (four items), psychological (four items), and urogenital (three items) symptoms. Each item is rated on a 5-point Likert scale ranging from 0 (no symptoms) to 4 (very severe symptoms). The total score ranges from 0 to 44. While the MRS originally categorized the total scores into four levels (no/little, 0–4; mild, 5–8; moderate, 9–16; severe, ≥17), this study dichotomized the scores into no/mild symptoms (0–8 points) and moderate/severe symptoms (9–44 points) to more effectively identify women experiencing significant menopausal symptoms. The internal consistency of the MRS in our sample was shown by a Cronbach’s α of .83.

#### Physical characteristics

Physical characteristics were assessed through comprehensive body measurements and physical fitness assessments.

Body measurements included height (cm), weight (kg), BMI (kg/m²), body fat (%), skeletal muscle mass (kg), waist circumference (cm), systolic and diastolic blood pressure (mmHg), and resting heart rate (bpm).

Physical fitness assessments included relative grip strength (kg; measured by taking the average of the left and right hands, with subsequent normalization by body weight [35]) and cardiorespiratory endurance (maximal oxygen consumption, VO₂max; assessed using a cycle ergometer).

#### Psychological characteristics

Psychological factors included psychological well-being and loneliness.

Psychological well-being: the Psychological Well-Being Scale (PWBS) [36] consists of 18 items rated on a 1–5 scale. The mean score was calculated across all items, with higher scores indicating greater psychological well-being. In this study, we used a Korean version translated by the authors. Cronbach’s α for the PWBS was .82.

Loneliness: the Loneliness and Social Isolation Scale (LSIS) [37] was developed in Korean and consists of six items across three subdomains: loneliness, social support, and social network (two items per subdomain). Each item is rated on a 0–3 scale. The mean scores of each subdomain were calculated and used as separate predictors in the analysis, with higher scores indicating greater loneliness, lower social support, and smaller social networks, respectively. Cronbach’s α for the LSIS in our sample was .69.

#### Lifestyle characteristics

Lifestyle factors included physical activity, dietary habits, routine activities, social relationships, and health behaviors.

Physical activity was measured over 8 days using wearable devices (Fitbit Charge 5, Fitbit Inc.), recording average daily steps and time spent in sedentary, light, moderate, and vigorous activities. Additionally, participants self-reported their number of days performing strength-training exercises in the past week.

Dietary habits were assessed using two components. Health-conscious dietary behaviors were measured using eight items developed by our research team based on the Seoul Food Survey [38]. Each item was rated on a 5-point scale from strongly agree (1) to strongly disagree (5). The items were “I control my food intake to maintain appropriate body weight,” “I consider health when I eat,” “I eat three times a day,” “I consider nutritional value when eating foods,” “I check food labels when purchasing processed foods,” “I try to eat variety of foods,” “I cook food in portions enough for one meal,” and “I often eat after 10 PM.” All items except the last one were reverse-coded so that higher total scores indicate more health-conscious eating habits (range, 8–40). The overall scale demonstrated acceptable internal consistency (Cronbach’s α=.70). Food consumption frequency was assessed for 10 food groups (grains, meat and eggs, fish, soy products, vegetables, dairy products, fruits, greasy food, snacks and sweets, and caffeine-containing beverages) and categorized as ≤1 time/week, 2–4 times/week, or ≥5 times/week.

Routine activities comprised household labor and sleep patterns. Household labor included time spent on meal preparation, laundry, house cleaning, financial management, shopping, and family care, measured in minutes per week through self-reported questionnaires. Sleep patterns were assessed by bedtime and wake-up time.

Social relationships encompassed family relationships and personal communication. Family relationships included the quality of relationships with spouse, children, parents, and in-laws, and the presence of family members with daily contact. Relationship quality was measured on a 1–6 scale and categorized into four groups: no applicable family member, slightly close (1, 2), moderate (3, 4), and good (5, 6), considering both the presence of the family member and the quality of the relationship. Time spent on personal communication was measured in minutes per day.

Health behaviors included smoking behavior, problematic drinking, and use of antihypertensive medication.

#### General characteristics

General characteristics included age (years), age at menarche (years), number of pregnancies, and number of chronic diseases, residential areas at the age of 13 years (Seoul, large cities, mid-sized cities, or rural), education level (high school or less, associate degree bachelor’s degree, or graduate degree), and current employment status (employed or unemployed). Monthly household income was quantified in five categories: <3 million Korean won (KRW; roughly 2,100 US dollars), 3 million–4.99 million KRW (roughly 2,100–3,499 US dollars), 5 million–6.99 million KRW (roughly 3,500–4,899 US dollars), 7 million–9.99 million KRW (roughly 4,900–6,999 US dollars), and ≥10 million KRW (roughly 7,000 US dollars).

### Study procedures

Participants visited a laboratory and completed a physical health questionnaire, body measurements, and physical fitness assessments. Participants then completed the self-report survey, after which they were provided with a wrist-worn wearable fitness tracker (Fitbit Charge 5) to monitor physical activity. Participants were instructed to wear the tracker for 8 consecutive days to record daily activity, including step counts and time spent at different intensity levels. Participants received 120,000 KRW (worth approximately 92 US dollars) in appreciation for their participation.

### Data analysis

The Random Forest technique was employed to identify significant predictors of menopausal symptoms among middle-aged women. This method is well-suited for datasets with many predictors relative to sample size and helps reduce overfitting [27]. The analysis comprised data preprocessing, model development, and performance evaluation. The dataset (N=94) had minimal missing data, with only 0.49% missing values among predictors. Analysis of missing patterns showed that 83 participants had complete data, while most incomplete cases lacked only 1–2 values. Missing values in the training set were imputed using the rfImpute function in R, while those in the test set were replaced with corresponding means from the training set to prevent data leakage [39]. We split the dataset into training (n=67, 70%) and test (n=27, 30%) sets for model development and final performance evaluation, respectively. Predictor variables were initially selected based on a comprehensive literature review of physical, psychological, and lifestyle domains (Supplementary Tables 1, 2). Feature selection was then conducted using recursive feature elimination with cross-validation (RFE-CV) on the training set to reduce the number of predictors while maintaining model accuracy [40].

We developed the random forest model using 5-fold cross-validation with three repeats to address potential overfitting [41,42]. Given the class imbalance (more women experienced moderate-to-severe symptoms), five resampling methods were applied: no resampling, down-sampling, up-sampling, synthetic minority over-sampling technique (SMOTE), and random over-sampling examples (ROSE) [43-45]. Hyperparameter tuning was performed via a grid search, testing different numbers of trees and values of mtry (ranging from 1 to √p, where p is the number of predictors). The optimal configuration was selected based on cross-validated performance, with balanced accuracy as the main criterion to account for class imbalance. To address concerns regarding model selection and sample size, additional validation was performed using Lasso regression [46]. Lasso with cross-validation was used to determine the optimal regularization parameter and compare feature selection results with those of random forest.

Model performance evaluation on the test set utilized four metrics [47]: area under the receiver operating characteristic curve (AUC) to assess discriminative ability, accuracy to measure prediction correctness, and sensitivity and specificity to assess correct identification of women with and without symptoms, respectively. The aim was to achieve high AUC and accuracy while maintaining a balance between sensitivity and specificity. Variable importance was assessed using the Mean Decrease Gini index, which quantifies each predictor’s contribution to accuracy by summing decreases in node impurity across all trees [48]. Partial dependence plots were then generated to explore the relationships between important predictors and symptom severity. All analyses were performed in R ver. 4.3.3 (R Core Team) using the randomForest, caret, and pROC packages [49-51].

## Results

### General characteristics of study participants

In this study, 29 participants (30.9%) had no/mild menopausal symptoms (MRS ≤8), while 65 participants (69.1%) had moderate/severe symptoms (MRS ≥9). The mean age of participants was 50.13 years. As shown in Table 1, participants had an average age of 50.1±3.1 years and a BMI of 23.0±3.1 kg/m^2^, indicating a generally healthy weight status. On average, they recorded 10,830 steps per day, reflecting an active lifestyle, and reported moderate psychological well-being scores (3.58±0.42 out of 5.0). Most participants were well educated, with 70.2% holding college degrees or higher, and the majority (58.5%) were employed, reflecting a largely middle-class demographic.

### Feature selection

**Figure 1.**
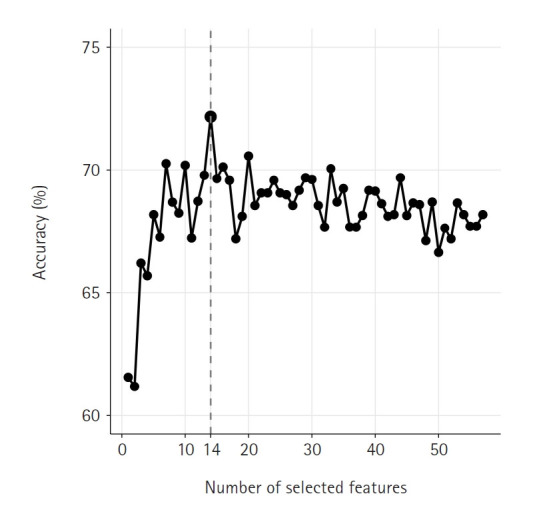
Model accuracy across different numbers of selected features using recursive feature elimination with cross-validation.

The RFE-CV method was used to identify the most relevant features for predicting menopausal symptoms. Features were selected to maximize model accuracy, defined as the proportion of correct predictions of both presence and absence of symptoms. As shown in Figure 1, the RFE-CV process selected 14 features from the initial 57 variables, optimizing model accuracy at approximately 72.2%. The selected features spanned demographic characteristics, physical health indicators, psychological factors, lifestyle behaviors, and dietary patterns. These features and their distributions by symptom severity are summarized in Table 2.

### Results of classification using random forest

The random forest model was developed using the 14 selected features and evaluated on the test set. Model development included hyperparameter tuning and evaluation of different resampling methods using 5-fold cross-validation with three repeats on the training set. Table 3 presents the cross-validated performance metrics for each resampling method, including 95% confidence intervals (CIs). No resampling achieved high AUC (85.2%; 95% CI, 80.0%–90.3%) and sensitivity (100.0%) but extremely low specificity (1.7%; 95% CI, 0%–4.9%), indicating poor prediction accuracy for women without symptoms. Down-sampling yielded comparable AUC (82.9%; 95% CI, 77.9%–87.9%) while achieving balanced sensitivity (73.2%; 95% CI, 65.1%–81.2%) and specificity (73.0%; 95% CI, 63.8%–82.2%). Up-sampling and SMOTE showed very high sensitivity (87.0% and 96.4%, respectively) but lower specificity (58.3% and 42.3%, respectively). ROSE improved specificity (81.3%; 95% CI, 73.4%–89.2%) but reduced sensitivity (57.9%; 95% CI, 48.8%–66.9%). Based on these comparisons, down-sampling was selected for final model development to ensure balanced predictive performance. Evaluation of the final model on the test set showed an accuracy of 74.1%, sensitivity of 78.9%, specificity of 62.5%, and AUC of 75.7%. These results suggest that the model achieved reasonably strong performance, particularly given the small sample size and class imbalance.

To further validate model selection, we conducted a comparative analysis using Lasso regression. As shown in Supplementary Figure 1, Lasso achieved a mean cross-validated accuracy of 0.72 at λ=0.097. It ultimately selected six predictors: age, psychological well-being, relative grip strength, relationship with spouse, vegetable consumption, and educational level. Importantly, these six predictors overlapped with the random forest RFE-CV results, reinforcing the role of both psychological and physical factors in menopausal symptoms. This concordance across methods strengthens confidence in our findings, particularly regarding the importance of psychological factors alongside physical characteristics. Although Lasso provided a more parsimonious model, we retained random forest due to its superior capacity to capture nonlinear relationships between predictors and menopausal symptoms [40,52].

### Feature importance

**Figure 2.**
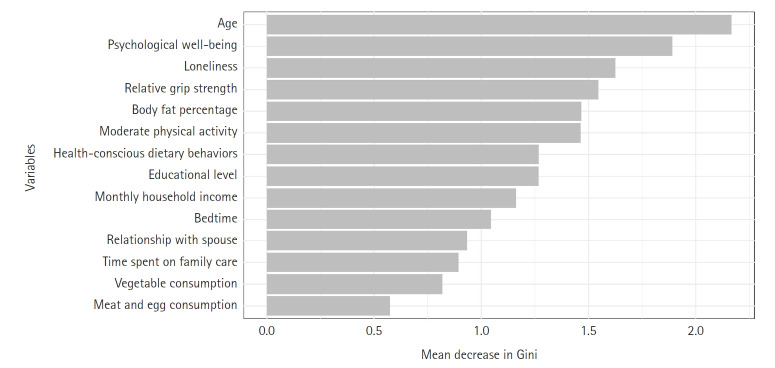
Variable importance plot of 14 predictors from random forest analysis.

Random forest analysis revealed the relative importance of the 14 predictors based on the Mean Decrease Gini measure (Figure 2). Age was the strongest predictor. Psychological factors were highly influential, with psychological well-being and loneliness ranking second and third. Among physical characteristics, relative grip strength and body fat percentage showed substantial importance. Lifestyle factors—including moderate physical activity and health-conscious dietary behaviors—demonstrated moderate importance, while bedtime, time spent on family care, relationship with spouse, and dietary frequencies (vegetable and meat/egg consumption) showed relatively lower importance. Sociodemographic variables, including educational level and household income, also displayed moderate importance.

### Partial dependence plots

**Figure 3.**
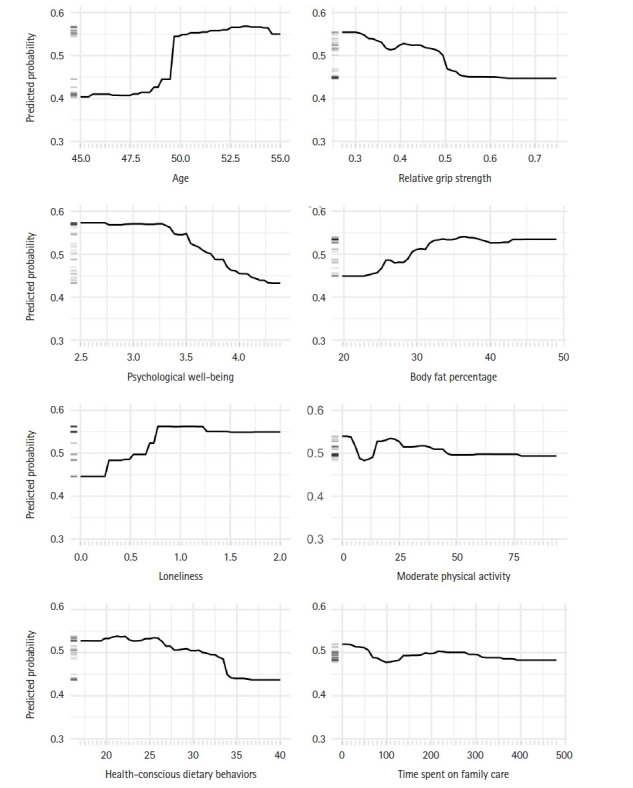
Partial dependence plots for key variables on menopausal symptoms.

Partial dependence plots were generated to examine the marginal effects of key predictors on the probability of moderate-to-severe symptoms, while holding other predictors at their average values (Figure 3). Age showed a threshold effect, with a sharp increase in symptom probability around age of 50 years. For psychological factors, psychological well-being maintained a stable relationship with symptom probability until a score of 3.5, after which a clear negative association was observed. Loneliness was strongly associated with increased symptom probability between scores of 0.5 and 1.0, after which the effect plateaued. Among physical factors, relative grip strength showed an inverse association with symptom probability, whereas body fat percentage demonstrated a positive relationship. For lifestyle factors, health-conscious dietary behaviors showed a notable negative association with symptom probability, while moderate physical activity and time spent on family care showed slight decreasing trends.

## Discussion

Our study demonstrated that the factors influencing menopausal symptoms are multifaceted, spanning physical, psychological, and lifestyle domains rather than being confined to hormonal changes alone. By applying a comprehensive machine learning approach, we identified a distinctive pattern of predictors that challenges traditional single-domain perspectives on menopause management. Using random forest analysis to simultaneously evaluate 57 potential variables across multiple domains, we generated a more nuanced understanding of the relative importance of diverse factors affecting symptom severity. The emergence of psychological, physical, and lifestyle variables as key predictors underscores the importance of adopting holistic, integrated approaches to menopause care. These findings provide an evidence-based foundation for the development of digital healthcare applications that address the interconnected nature of menopausal symptoms through comprehensive monitoring and targeted interventions.

Our analysis revealed that psychological factors were among the strongest predictors of menopausal symptoms. Psychological well-being ranked second in importance after age, with higher levels associated with a lower probability of experiencing moderate-to-severe symptoms. Although the causal direction requires further investigation, previous research has shown that psychological changes during menopause can lead to unhealthy behaviors such as binge eating or reduced physical activity [6], creating cycles that may exacerbate symptoms [16,17]. These findings suggest that digital healthcare solutions should incorporate comprehensive mental health features, including mood-tracking tools and personalized stress management coaching. Similarly, higher levels of loneliness were significantly associated with a greater likelihood of experiencing menopausal symptoms, consistent with prior studies [18], highlighting the potential value of digital features designed to foster social connection.

Physical characteristics, particularly relative grip strength and body fat percentage, also showed strong associations with symptom severity. Relative grip strength demonstrated a negative relationship with symptoms, consistent with previous findings that reduced muscle strength can limit participation in regular exercise [5,11]. This can contribute to a feedback loop in which diminished physical capacity reduces activity levels, thereby worsening symptoms. Likewise, body fat percentage was a significant predictor, consistent with earlier research linking higher adiposity to more severe menopausal symptoms [10]. These findings suggest the importance of integrating fitness-related features into digital interventions, with emphasis on strength training and body composition monitoring.

Health-conscious dietary behaviors and moderate physical activity also emerged as significant lifestyle predictors of menopausal symptoms. The role of diet is particularly notable, given evidence linking high intake of saturated fats and processed foods to psychological, vasomotor, and somatic symptoms [20]. Partial dependence plots indicated that higher levels of moderate physical activity were associated with a slight decrease in symptom probability, reinforcing previous evidence on the benefits of regular activity for symptom management [7]. Together, these results suggest that digital healthcare applications should include balanced guidance on nutrition and physical activity. Moreover, relationship quality with spouse and time spent on family care were identified as predictors in the random forest model, consistent with prior evidence showing that social support and family relationships mitigate menopausal challenges, with stronger ties associated with fewer and less severe symptoms [19].

Finally, sociodemographic factors such as educational level and monthly household income showed moderate associations with symptom severity. These findings align with previous studies reporting that women with lower educational attainment and household income are more likely to experience severe symptoms, including sleep disturbances and depressed mood [22,23]. Such results point to potential disparities in symptom burden and access to management resources across different social strata.

Taken together, our findings have several important implications for the development of effective digital healthcare solutions for menopausal symptom management. First, identifying the relative importance of predictors allows for the creation of more targeted and efficient digital interventions, enabling applications to prioritize monitoring and intervention features with the greatest potential impact. In particular, the results highlight the need to address psychological well-being and social support alongside physical health. Second, the wide range of predictors emphasizes the necessity of comprehensive digital solutions that integrate psychological support, activity tracking, nutrition guidance, and sleep management. Finally, these findings respond to a key limitation of existing menopause-focused digital healthcare applications, which often lack evidence-based frameworks, by providing empirical guidance for selecting critical monitoring and intervention components [19].

Based on the identified predictors, we propose that effective digital healthcare applications for menopausal symptom management should incorporate several specific components. For psychological well-being, which emerged as a highly important predictor, applications could include mood-tracking tools, guided mindfulness exercises, and cognitive-behavioral therapy (CBT) modules for stress management. Evidence indicates that mindfulness-based interventions and CBT are effective in alleviating hot flashes, insomnia, depression, and anxiety associated with menopause [53,54]. For physical health predictors such as relative grip strength and body fat percentage, applications should provide guided strength-training programs tailored to middle-aged women, as well as body composition tracking with personalized recommendations. For dietary behaviors, which showed moderate importance, applications could feature meal-planning tools, nutritional education designed for menopausal needs, and dietary tracking with feedback functions. Sleep management tools addressing bedtime patterns could include sleep-quality monitoring and individualized recommendations for optimal sleep schedules. Finally, features supporting family relationships and social connectedness could help mitigate loneliness and strengthen relationship factors identified in our analysis.

Our study employed several methodological approaches to ensure robust and reliable findings. We selected random forest analysis due to its advantages with smaller datasets. Tree-based ensemble methods such as the random forest are relatively robust against overfitting even with limited samples, as they reduce variance by averaging multiple decision trees and effectively capture nonlinear relationships without distributional assumptions [55]. We confirmed the robustness of feature selection by comparing results with Lasso regression, which identified overlapping key predictors across psychological, physical, and lifestyle domains. We also carefully addressed data quality concerns that might influence model reliability. The dataset (N=94) had minimal missing data (0.49%), and no systematic patterns were detected, suggesting that missingness was approximately completely at random. Given the low percentage and random distribution of missing data, it is unlikely that our imputation method introduced meaningful bias in variable importance rankings.

Despite these strengths, several limitations must be acknowledged. The relatively small sample size and focus on middle-class women in Seoul, Korea, limit the generalizability of findings to other populations. To mitigate the effects of sample size limitations, we employed cross-validation, multiple resampling techniques to address class imbalance, and sensitivity analyses with alternative feature-selection methods. Nevertheless, future research with larger, more diverse populations is needed to validate these results and potentially identify additional relevant factors. In addition, the cross-sectional design precludes causal inferences regarding relationships between predictors and menopausal symptoms. Although random forest analysis is effective for identifying influential predictors, it has limited capacity to explain underlying mechanisms, particularly for categorical variables. Longitudinal studies that track women’s experiences over time would help clarify temporal dynamics and causal directions among psychological well-being, lifestyle factors, and menopausal symptoms.

This study provides comprehensive evidence for the multifaceted nature of menopausal symptoms, highlighting the importance of physical, psychological, and lifestyle predictors in shaping symptom severity. In particular, psychological well-being and lifestyle behaviors emerged as crucial factors, underscoring the need for holistic approaches to symptom management. Future research should investigate interactions among these domains using intervention trials and advanced analytical methods, such as structural equation modeling. Such work would contribute to the development of more effective and personalized strategies for managing menopausal symptoms, ultimately improving women’s health and quality of life during this critical life transition.

## Figures and Tables

**Table 1.** Descriptive characteristics of participants (N=94)

Variable	Categories	Mean±SD or n (%)
Menopausal rating scale	Total	13.18±6.69
	No/mild (0–8)	29 (30.9)
	Moderate/severe (9–44)	65 (69.1)
Body mass index (kg/m²)		22.95±3.07
Steps per day		10,829.82±4,542.83
Psychological well-being		3.58±0.42
Age (year)		50.13±3.13
Age at menarche (year)		13.66±1.34
Number of pregnancies		1.96±1.03
Educational level	High school or less	14 (14.9)
	Associate degree	14 (14.9)
	Bachelor’s degree	58 (61.7)
	Graduate degree	8 (8.5)
Employment status	Employed	55 (58.5)
	Unemployed	39 (41.5)
Monthly household income (million KRW)	<3	7 (7.5)
	3–4.99	11 (11.7)
	5–6.99	23 (24.5)
	7–9.99	28 (29.8)
	≥10	25 (26.6)

KRW: Korean won (1 million KRW is approximately 700 US dollars).

**Table 2.** Characteristics of Selected Predictive Variables by Menopausal Symptom Severity (N=94)

Variable	Categories	Mean±SD or n (%)		*t*/χ^2^	*p*
		Moderate/severe (n=65)	No/mild (n=29)		
Physical characteristics					
Relative grip strength		0.40±0.07	0.44±0.09	−2.28	.025
Body fat percentage		33.00±5.72	30.68±5.94	1.80	.076
Psychological characteristics					
Psychological well-being		3.50±0.41	3.76±0.36	−2.92	.004
Loneliness		0.86±0.71	0.59±0.67	1.75	.083
Lifestyle characteristics					
Moderate physical activity		20.47±16.87	19.26±19.09	0.31	.758
Time spent on family care		67.76±169.00	121.72±199.00	−1.35	.180
Health-conscious dietary behaviors		25.99±4.08	28.34±5.08	−2.39	.019
Vegetable consumption (time/week)	≤1	4 (6.1)	2 (6.90)	3.63	.163
	2–4	36 (55.4)	10 (34.5)		
	≥ 5	25 (38.5)	17 (58.6)		
Meat and egg consumption (time/week)	≤ 1	2 (3.1)	0 (0)	3.61	.164
	2–4	31 (47.7)	9 (31.0)		
	≥5	32 (49.2)	20 (69.0)		
Relationship with spouse	Single/divorced	4 (6.2)	1 (3.5)	2.30	.513
	Poor	5 (7.7)	3 (10.3)		
	Average	17 (26.2)	4 (13.8)		
	Good	39 (60.0)	21 (72.4)		
Bedtime	22:00–22:59	1 (1.54)	6 (20.7)	13.16	.011
	23:00–23:59	18 (27.7)	8 (27.6)		
	00:00–00:59	23 (35.4)	7 (24.1)		
	01:00–01:59	10 (15.4)	6 (20.7)		
	After 02:00	13 (20.0)	2 (6.9)		
General characteristics					
Age (year)		50.80±2.79	48.62±3.35	3.28	.002
Educational level	High school or less	8 (12.3)	6 (20.7)	5.20	.158
	Associate degree	8 (12.3)	6 (20.7)		
	Bachelor’s degree	45 (69.2)	13 (44.8)		
	Graduate degree	4 (6.2)	4 (13.8)		
Monthly household income (million KRW)	<3	6 (9.2)	1 (3.5)	5.53	.237
	3–4.99	5 (7.7)	6 (20.7)		
	5–6.99	14 (21.5)	9 (31.0)		
	7–9.99	21 (32.3)	7 (24.1)		
	≥10	19 (29.2)	6 (20.7)		

KRW: Korean won (1 million KRW is approximately 700 US dollars).

**Table 3.** Cross-validation performance of the random forest classification model using five different sampling methods

Sampling method	mtry	ntree	AUC (95% CI)^†^	Sensitivity (95% CI)^†^	Specificity (95% CI)^†^
None	1	1,000	85.2 (80.0–90.3)	100.0 (100.0–100.0)	1.7 (0.0–4.9)
Down-sampling	1	500	82.9 (77.9–87.9)	73.2 (65.1–81.2)	73.0 (63.8–82.2)
Up-sampling	1	500	83.0 (77.4–88.5)	87.0 (83.9–90.0)	58.3 (45.8–70.8)
SMOTE	1	1,000	83.2 (77.2–89.2)	96.4 (93.8–99.1)	42.3 (29.8–54.9)
ROSE	3	500	80.5 (74.3–86.6)	57.9 (48.8–66.9)	81.3 (73.4–89.2)

AUC, Area under the receiver operating characteristic curve; CI, confidence interval; mtry, number of variables randomly sampled at each split in the random forest model; ntree, Number of trees grown in the random forest model; ROSE, random over-sampling examples; SMOTE, synthetic minority over-sampling technique.

† 95% CIs calculated from 5-fold cross-validation with three repeats.

## Supplementary materials

Further details on supplementary materials are presented online (available at https://doi.org/10.4069/whn.2025.08.12).

Supplementary Table 1. Descriptive statistics of continuous variables in the study (N=94)

Supplementary Table 2. Descriptive statistics of categorical variables in the study (N=94)

Supplementary Figure 1. Lasso regression model validation results. (A) Cross-validated accuracy across different regularization parameters (lambda). The vertical red dashed line indicates the optimal lambda value (0.097) that maximizes prediction accuracy. (B) Relationship between lambda values and the number of non-zero coefficients retained in the model. The optimal model at lambda=0.097 (vertical red dashed line) retains six predictors.
