# Supplementary material for: Exploring the relative importance of the factors associated with menopausal symptoms using a random forest model: a cross-sectional study
Source: Womens Health Nurs. 2025 Sep 30;31(3):227–40. doi: 10.4069/whn.2025.08.12 (PMC12558644; doi:10.4069/whn.2025.08.12)
Supplement: Supplementary Table 1. — Descriptive statistics of continuous variables in the study (N=94) [file whn-2025-08-12-Supplementary-Table-1.pdf]

Supplementary Table 1. Descriptive statistics of continuous variables in the study (N=94)

Variable	Mean ± SD		t	*p*
	Moderate/severe (n = 65)	No/mild (n = 29)		
Physical characteristics				
Height (cm)	160.23 ± 4.82	160.57 ± 5.82	−0.29	.770
Weight (kg)	59.48 ± 8.35	57.83 ± 8.28	0.89	.378
Body mass index (kg/m^2)	23.19 ± 3.18	22.39 ± 2.78	1.17	.246
Body fat (%)	33.00 ± 5.72	30.68 ± 5.94	1.80	.076
Skeletal muscle mass (kg)	21.18 ± 2.22	21.31 ± 2.69	−0.24	.808
Waist circumference (cm)	78.66 ± 9.34	76.25 ± 7.15	1.24	.220
Systolic blood pressure (mmHg)	122.83 ± 14.15	125.90 ± 18.57	−0.88	.382
Diastolic blood pressure (mmHg)	74.95 ± 9.91	77.97 ± 11.63	−1.29	.201
Resting heart rate (beats/min)	77.56 ± 9.36	77.97 ± 11.63	0.06	.945
Relative grip strength	0.40 ± 0.07	0.44 ± 0.09	−2.28	.025
Cardiorespiratory endurance (VO_{2max})	23.32 ± 3.43	24.91 ± 4.27	−1.93	.057
Psychological characteristics				
Psychological well-being	3.50 ± 0.42	3.76 ± 0.36	−2.92	.004
Loneliness	0.86 ± 0.71	0.59 ± 0.67	1.75	.083
Social support	1.02 ± 0.81	1.03 ± 0.67	-0.10	.917
Social network	0.90 ± 0.49	0.84 ± 0.54	0.47	.643
Lifestyle characteristics				
Steps per day	10,517.23 ± 4,209.45	11,490.79 ± 5,218.63	−0.96	.341
Sedentary time (min/day)	632.26 ± 180.75	653.47 ± 149.23	−0.55	.582
Light physical activity (min/day)	240.18 ± 64.50	250.76 ± 67.65	−0.72	.472
Moderate physical activity (min/day)	20.47 ± 16.88	19.26 ± 19.09	0.31	.758
Vigorous physical activity (min/day)	25.99 ± 20.99	32.23 ± 23.49	−1.31	.193
Number of days of strength training	0.56 ± 1.24	0.72 ± 1.44	−0.56	0.579
in the past week	0.56 ± 1.24	0.72 ± 1.44	−0.56	.579
Health-conscious dietary behaviors	25.99 ± 4.08	28.34 ± 5.08	−2.39	.019
Time spent on meal preparation (min/week)	544.15 ± 422.91	568.28 ± 395.50	−0.26	.795
Time spent on laundry (min/week)	139.25 ± 124.35	181.03 ± 182.41	−1.12	.269
Time spent on house cleaning (min/week)	188.45 ± 152.39	238.97 ± 222.99	−1.11	.274
Time spent on financial management (min/week)	44.38 ± 47.85	63.62 ± 86.05	−1.13	.267
Time spent on shopping (min/week)	140.08 ± 158.33	162.76 ± 227.12	−0.49	.629
Time spent on family care (min/week)	67.76 ± 169.00	121.72 ± 199.00	−1.35	.180
Time spent on personal communication (min/day)	49.63 ± 62.77	50.17 ± 42.01	−0.05	.961
Problematic drinking	2.62 ± 3.09	1.93 ± 2.14	1.24	.219
General characteristics				
Age (year)	50.80 ± 2.79	48.62 ± 3.35	3.28	.002
Age at menarche (year)	13.66 ± 1.42	13.66 ± 1.14	0.00	.997
Number of pregnancies	1.94 ± 1.16	2.00 ± 0.65	−0.33	.744
Number of chronic diseases	0.83 ± 1.07	0.52 ± 0.74	1.64	.104
