# Supplementary material for: Exploring the relative importance of the factors associated with menopausal symptoms using a random forest model: a cross-sectional study
Source: Womens Health Nurs. 2025 Sep 30;31(3):227–40. doi: 10.4069/whn.2025.08.12 (PMC12558644; doi:10.4069/whn.2025.08.12)
Supplement: Supplementary Table 2. — Descriptive statistics of categorical variables in the study (N=94) [file whn-2025-08-12-Supplementary-Table-2.pdf]

Supplementary Table 2. Descriptive statistics of categorical variables in the study (N=94)

Variable	Categories	n (%)		χ^2	*p*
		Moderate/severe (n = 65)	No/mild (n = 29)		
Lifestyle characteristics					
Dietary habits (time/week)					
Grain	≤ 1	2 (3.1)	1 (3.4)	1.82	.402
	2–4	14 (21.5)	10 (34.5)		
	≥ 5	49 (75.4)	18 (62.1)		
Meat and egg	≤ 1	2 (3.1)	0 (0)	3.61	.164
	2–4	31 (47.7)	9 (31.0)		
	≥ 5	32 (49.2)	20 (69.0)		
Fish	≤ 1	41 (63.1)	19 (65.5)		
	2–4	22 (33.9)	10 (34.5)		
	≥ 5	2 (3.1)	0 (0)		
Soy products	≤ 1	12 (18.5)	5 (17.2)	0.70	.704
	2–4	44 (67.7)	18 (62.1)		
	≥ 5	9 (13.9)	6 (20.7)		
Vegetable	≤ 1	4 (6.2)	2 (6.9)	3.63	.163
	2–4	36 (55.4)	10 (34.5)		
	≥ 5	25 (38.5)	17 (58.6)		
Dairy products	≤ 1	23 (35.4)	7 (24.1)	1.73	.421
	2–4	25 (38.5)	11 (37.9)		
	≥ 5	17 (26.2)	11 (37.9)		
Fruits	≤ 1	7 (10.8)	2 (6.9)	0.37	.833
	2–4	31 (47.7)	14 (48.3)		
	≥ 5	27 (41.5)	13 (44.8)		
Greasy food	≤ 1	11 (16.9)	4 (13.8)	0.18	.915
	2–4	42 (64.6)	19 (65.5)		
	≥ 5	12 (18.5)	6 (20.7)		
Snacks and sweets	≤ 1	20 (30.8)	7 (24.1)	0.65	.724
	2–4	35 (53.9)	16 (55.2)		
	≥ 5	10 (15.4)	6 (20.7)		
Caffeine-containing beverages	≤ 1	5 (7.7)	1 (3.5)	0.62	.732
	2–4	10 (15.4)	5 (17.2)		
	≥ 5	50 (76.9)	23 (79.3)		
Routine activities					
Bedtime	22:00–22:59	1 (1.5)	6 (20.7)	13.16	.011
	23:00–23:59	18 (27.7)	8 (27.6)		
	00:00–00:59	23 (35.4)	7 (24.1)		
	01:00–01:59	10 (15.4)	6 (20.7)		
	After 02:00	13 (20.0)	2 (6.9)		
Wake-up time	Before 06:00	4 (6.2)	7 (24.1)	7.67	.104
	06:00–06:59	18 (27.7)	6 (20.7)		
	07:00–07:59	22 (33.8)	11 (37.9)		
	08:00–08:59	13 (20.0)	3 (10.3)		
	09:00–11:00	8 (12.3)	2 (6.9)		

(Continued to the next page)

Supplementary Table 2. Continued

Variable	Categories	n (%)		χ^2	*p*
		Moderate/severe (n = 65)	No/mild (n = 29)		
Social relationships					
Daily contact with family members	Yes	58 (89.2)	26 (89.7)	0.00	.951
	No	7 (10.8)	3 (10.34)		
Relationship with spouse	Single/divorced	4 (6.2)	1 (3.5)	2.30	.513
	Poor	5 (7.7)	3 (10.3)		
	Average	17 (26.2)	4 (13.8)		
	Good	39 (60.0)	21 (72.4)		
Relationship with children	No children	6 (9.23)	1 (3.5)	2.13	.546
	Poor	2 (3.08)	0 (0)		
	Average	14 (21.5)	8 (27.6)		
	Good	43 (66.2)	20 (69.0)		
Relationship with parents	No living parent	8 (12.3)	3 (10.3)	2.42	.489
	Poor	10 (15.4)	2 (6.9)		
	Average	29 (44.6)	12 (41.4)		
	Good	18 (27.7)	12 (41.4)		
Relationship with in-laws	No living in-law	18 (27.7)	7 (24.1)	1.54	.674
	Poor	15 (23.1)	6 (20.7)		
	Average	28 (43.1)	12 (41.4)		
	Good	4 (6.2)	4 (13.8)		
Health behaviors					
Smoking behavior	Smoker	2 (3.1)	0 (0)	0.92	.340
	Non-smoker	63 (96.9)	29 (100)		
Use of antihypertensive medication	Yes	6 (9.2)	4 (13.8)	0.44	.508
	No	59 (90.8)	25 (86.2)		
General characteristics					
Place of residence during elementary school	Seoul	34 (52.3)	18 (62.2)	1.93	.587
	Large cities	12 (18.5)	4 (13.8)		
	Mid-sized cities	4 (6.2)	3 (10.3)		
	Rural	15 (23.1)	4 (13.8)		
Educational level	High school or less	8 (12.3)	6 (20.7)	5.20	.158
	Associate degree	8 (12.3)	6 (20.7)		
	Bachelor's degree	45 (69.2)	13 (44.8)		
	Graduate degree	4 (6.2)	4 (13.8)		
Employment status	Employed	39 (60.0)	13 (55.3)	0.19	.661
	Unemployed	26 (40.0)	16 (44.8)		
Monthly household income (million KRW)	< 3	6 (9.2)	1 (3.5)	5.53	.237
	3–4.99	5 (7.7)	6 (20.7)		
	5–6.99	14 (21.5)	9 (31.0)		
	7–9.99	21 (32.3)	7 (24.1)		
	≥ 10	19 (29.2)	6 (20.7)		

KRW: Korean won (1 million KRW is approximately 700 US dollars).

https://doi.org/10.4069/whn.2025.08.12
