# Supplementary material for: Exploring the relative importance of the factors associated with menopausal symptoms using a random forest model: a cross-sectional study
Source: Womens Health Nurs. 2025 Sep 30;31(3):227–40. doi: 10.4069/whn.2025.08.12 (PMC12558644; doi:10.4069/whn.2025.08.12)
Supplement: Supplementary Figure 1. — Lasso regression model validation results. (A) Cross-validated accuracy across different regularization parameters (lambda). The vertical red dashed line indicates the optimal lambda value (0.097) that maximizes prediction accuracy. (B) Relationship between lambda values and the number of non-zero coefficients retained in the model. The optimal model at lambda=0.097 (vertical red dashed line) retains six predictors. [file whn-2025-08-12-Supplementary-Figure-1.pdf]

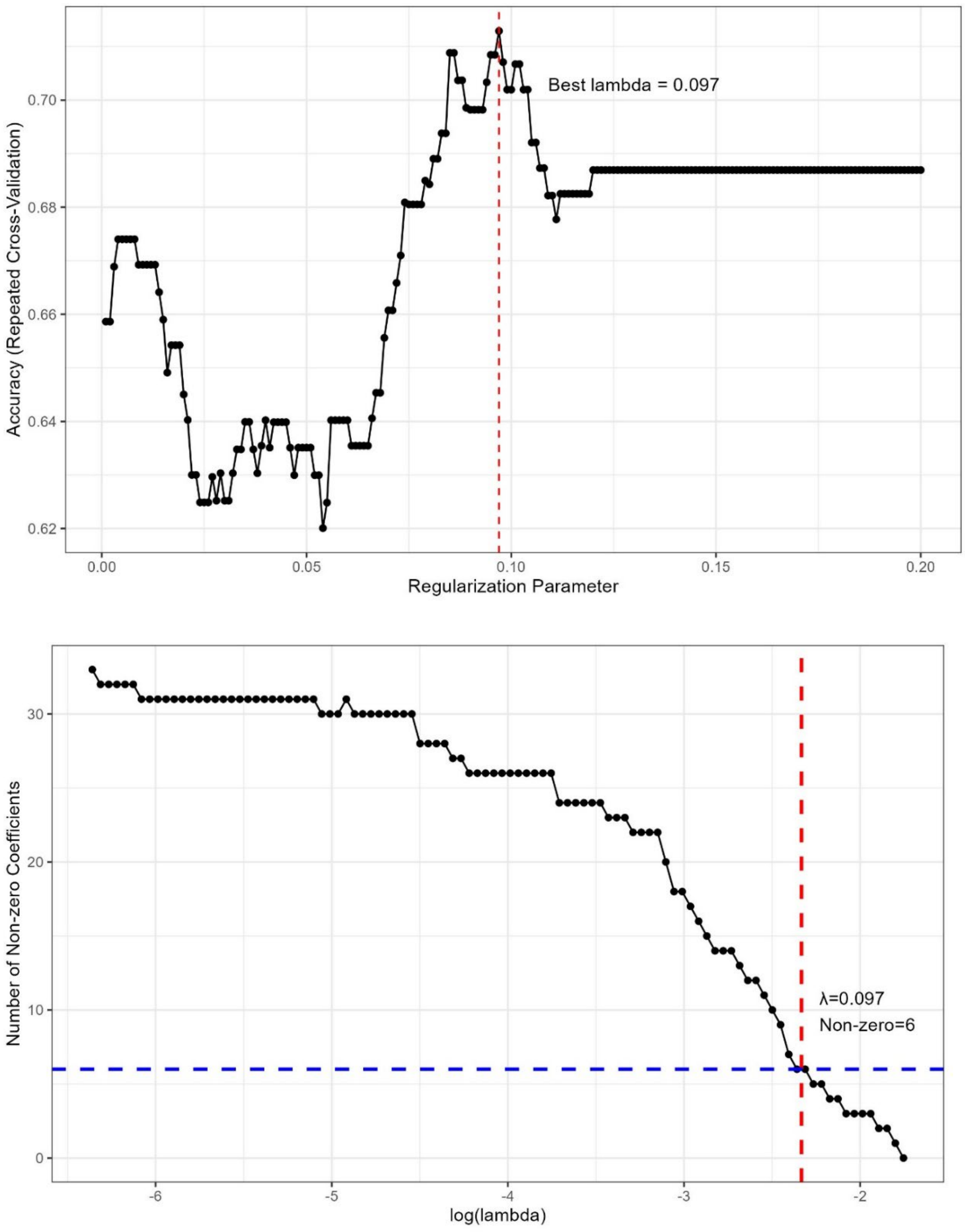

Supplementary Figure 1. Lasso regression model validation results. (A) Cross-validated accuracy across different regularization parameters (lambda). The vertical red dashed line indicates the optimal lambda value (0.097) that maximizes prediction accuracy. (B) Relationship between lambda values and the number of non-zero coefficients retained in the model. The optimal model at lambda=0.097 (vertical red dashed line) retains six predictors.
